# Societal Criticism towards COVID-19: Assessing the Theory of Self-Diagnosis Contrasted to Medical Diagnosis

**DOI:** 10.3390/diagnostics11101777

**Published:** 2021-09-27

**Authors:** Dimitra S. Mouliou, Ioannis Pantazopoulos, Konstantinos I. Gourgoulianis

**Affiliations:** 1 Department of Emergency Medicine, Faculty of Medicine, University of Thessaly, BIOPOLIS, 41110 Larissa, Greece; pantazopoulosioannis@yahoo.com; 2 Department of Respiratory Medicine, Faculty of Medicine, University of Thessaly, BIOPOLIS, 41110 Larissa, Greece; kgourg@uth.gr

**Keywords:** COVID-19, diagnosis, self-diagnosis, autodiagnosis, self-tests, physicians, emergency

## Abstract

Background: Coronavirus disease 2019 (COVID-19) has emerged as a pandemic introducing the mass autodiagnosis via rapid antigen testing methods, and self-tests were important for several populaces, yet with several neglected issues. In addition, hospital diagnosis was a target of many people or media, as the various COVID-19 clinical phenotypes trammel the precise emergency physicians’ response. Methods: A web-based questionnaire was disseminated through social media in the first half of August 2021 in the Greek populace, assessing the societal criticism for autodiagnosis and medical diagnosis and their issues, just before the occurrence of the fourth pandemic wave in the country. Results: Two thirds of the responders characterized self-tests as unreliable and two fifths reported them dangerous. Reliability (OR 1.335; CI 0.060–0.300; *p* = 0.000) and danger (OR 5.068; CI 3139–8184; *p* = 0.000) were significant predictors for the population-based sample’s volition for a self-test. Reversely, regarding medical diagnosis, half of the responders reported the lack of reliability and effectiveness in the emergency departments, which had a significant impact on willingness to visit a hospital if needed (OR 3.207; CI 1987–5182; *p* = 0.000 and OR 3.506; CI 2167–5670; *p* = 0.000). Conclusions: The importance of community-based questionnaires is highlighted for assessing people’s criticism and improving the highlighted points in several topics.

## 1. Introduction

Coronaviruses have emerged as an ultimatum for populaces since the early beginning of the 21st century. In December 2019, a novel Severe Acute Respiratory Syndrome Coronavirus 2 (SARS-CoV-2) was identified from a cluster of cases of pneumonia in Wuhan, China [1]. On 30 January 2020, the World Health Organization (WHO) announced the Coronavirus Disease 19 (COVID-19) as a Public Health Emergency of International Concern, and a month and a half later, the COVID-19 epidemic was portrayed as a pandemic [2]. Heretofore, epidemiological, emergency and respiratory scientific communities have exerted numerous endeavours to surveil, superintend and cure confirmed COVID-19 cases.

Doubtlessly, the COVID-19 pandemic has turned the health care system upside down and has challenged people’s sense of precariousness of diagnosing the virus. General hospitals, private clinics and community physicians continue to diagnose SARS-CoV-2 infections so as to combat COVID-19. Especially in summer days, some countries were believed to be in the fourth wave of pandemic spread, in parallel with vaccination policies as well as the occurrence of new viral mutants [3,4,5]. Europe is struggling for balance from the midst of the summer, and, currently, Greece has been facing the fourth wave since late August.

In 2000, Greece counted 337 general hospitals, but this number is steadily falling, and until now, the country has about two thirds of the hospitals that were recorded in the beginning of this century [6]. In addition, Greece counts two university departments of emergency medicine included in the hospitals’ overall figures. Although there has been a recorded decrease in the number of healthcare institutions in Greece, the number of their personnel was fluctuating in the first decade and has been declining ever since [6]. Indubitably, all emergency clinics had been the face of all the hospitals, which initially came to blows especially over COVID-19 critical cases during the pandemic, and peculiarly in a country where the rates of risk factors for a severe COVID-19 illness are extremely high [7]. However, community physicians contributed to the pandemic, especially regarding mild-to-moderate COVID-19 cases that did not require hospital and intensive care units. Generally, the country counts about 6 physicians per 1000 citizens [8]. Greece is considered to be a country with an overproduction of clinical doctors and experts, but unemployment rates are increasing in parallel with the Greek financial crisis.

Antigen tests were commonly used in the diagnosis of respiratory pathogens, such as influenza viruses and respiratory syncytial virus, even before the emergence of SARS-CoV-2. The U.S. Food and Drug Administration (FDA) has granted emergency use authorization (EUA) for antigen tests that can identify SARS-CoV-2 [9]. COVID-19 is the first pandemic wherein self-diagnosis has been partially actualized via rapid diagnostic tests. Globally, America was the first to follow this concept, and regarding Europe, Greece was the first country to introduce free-of-cost rapid tests, available in pharmacies for the overall populace for autodiagnosis purposes and determined time in late March. From April, the Greek authorities declared self-testing mandatory for public sector and specific private sector services’ employees, who should register the result in the government self-testing platform. Everyone with a social security number was entitled to four of the test kits per month, as well as people on holidays. Some people also tried COVID-19 symptoms’ online applications in parallel with self-testing. Especially, unvaccinated students should take two self-test per week for the coming school year in Greece. However, the false test results occurring in all testing strategies, and their various sensitivities, led to a societal doubt for the self-tests’ reliability [10]. In addition, there arose several other issues for self-testing, concerning the result’s privacy as a medical event, accuracy, safety and economic or other environmental parameters.

This is the first study to assess the theory of autodiagnosis via rapid antigen testing and its parameters, through societal criticism and attitudes during the COVID-19 pandemic, as revealed from an online survey via a Web-based Questionnaire (WBQ). Furthermore, societal estimates about medical diagnosis, compared to autodiagnosis, are presented. A final comparison between self-diagnosis and medical diagnosis, through the societal point of view, for the COVID-19 pandemic is presented.

## 2. Materials and Methods

### 2.1. WBQ Design

Social networks have fostered public gaze to a fabulous extent, thus serving a sophisticated target for monitoring society. Although WBQs are currently considered as a fluid form of observational, descriptive and analytical studies, they bespeak an upcoming propitious tool, accrediting experts to combine ontological, ethical and epistemological principles, to surveil society. WBQs enable motivated individuals to provide their answers, rapidly at the touch of a button, and they are automated, cost-effective and error-free [11,12]. The traditional closed-ended WBQs, structured with qualitative categorical or dichotomous questions, seem to be advantageous psychometric attempts and are desirable options for participation, contrary to open-ended questions requiring written answers [13].

The WBQ of the study consisted primarily of binary questions for demographics such as gender and level of education ((i) mandatory education or university and (ii) master of science or doctor of philosophy). Considering the qualitative type of the WBQ, for a better e-sample response—in addition to the fact that European countries are mainly aging—the concept of age-related questions was to follow a generation-based model with age ranges to reveal each generation’s criticism and attitudes. Generation categories included (i) Baby Boomers (age range 57–75 in 2021); (ii) Generation X (age range 41–56 in 2021); (iii) Millennials (age range 25–40 in 2021); and (iv) Generation Z, restricted to adults (18–24 in 2021). The exclusion criteria for WBQs were those under 18 and above 75 years of age.

The aim of the survey was to compare self-diagnosis with medical diagnosis, so the concept of the WBQ was to provide the same the same questions for self-diagnosis as well as medical diagnosis in order to make an actual comparison. Since most community physicians sent severe and critical cases to hospitals, and as EDs were doubtlessly the face of the hospitals that managed the COVID-19 pandemic at its first stages, the comparison especially targeted hospital emergency physicians. In such manner, 5-Likert type questions comprised the WBQ, including self-testing and medical reliability, effectiveness, management and risk of exposure to SARS-CoV2 or chemical risks (for self-testing). In addition, since this pandemic time was the first use of rapid testing for autodiagnosis purposes at a global level, the same questions for anxiety before testing, the cost and further self-treatment and self-testing when vaccinated and exposed to COVID-19 cases were included. A question regarding whether someone had performed a rapid test for autodiagnosis was also included. People who had never performed self-testing were excluded from the survey, so as to have an accurate crisis only from the people who have utilized self-tests. Finally, 2 basic binary questions were included for the medical and autodiagnosis comparison regarding where someone would do without force a rapid test for self-testing or not, when needed, and whether someone would go to the hospital immediately when urgent or to a community private physician.

The other important aim was to monitor the environmental issues that occur with self-testing, which have never been examined in this ongoing pandemic. For that purpose, categorical and binary questions were included in the survey, concerning the management of self-testing waste materials, the gloves during testing, the interfacing with rapid test liquids and the room where autodiagnosis takes place.

### 2.2. Population-Based Sample

The survey was conducted in the Greek mainland, where strict lockdown policies were imposed and where the populace was the first to have cost-free self-tests for some time. Self-testing was mandatory for specific employees, and the assessment would be conducted by people who have already used self-tests for several months. Greece is also facing an ongoing financial crisis that has affected hospitals, and thus it was important to reveal a societal review about their efficacy in this pandemic so as to improve in the next epidemics. Finally, Greece is the sole country that performs such daily rapid testing to monitor COVID-19, but doubtlessly, such strategies may lead to false results [10] and people would objectively judge the rapid tests’ overall issues.

### 2.3. WBQ Administration

Adults were randomly invited to participate in the survey through social media shares and announcements. Informed consent was obtained from all subjects during accepting participation in the study. WBQs were submitted in Google forms, and data were saved in an Excel spreadsheet.

### 2.4. Statistical Analysis

Statistical analyses were effectuated via the statistical software IBM SPSS statistics for Windows, Version 26.0 (headquartered in Chicago). Cronbach’s alpha coefficient was applied for internal consistency reliability. Data normality was assessed with Kolmogorov–Smirnov test. Tests were two-tailed, and the level of statistical significance was established at *p* ≤ 0.05. Chi-square test was applied for comparisons of frequencies, and Bonferroni correction was used for comparisons between subgroups. Spearman coefficient was used to evaluate correlations between variables. Mann–Whitney-U test and Kruskal–Wallis-H test were used for estimates of mean ranks among subgroups. Logistic regression analysis was considered for the investigation of potential predictors of volition to self-testing and Emergency Departments (EDs) for medical diagnosis.

## 3. Results

### 3.1. The Distribution of Genders, Generations and Education Levels

The population-based sample consisted of 268 men (43.6%) and 346 women (56.4%). As regards the generations, there were 62 Generation Z (10.1%), 216 Millennials (35.2%), 287 Generation X (46.7%) and 49 Baby Boomers (8%) counted in total. Table 1 describes the distribution of genders and their education levels across the different generations. 

There was no significant difference for genders among generations, but concerning the education level, most Generation Z and Millennials reported a further university education above the basic schooling (29% vs. 71%, *p* = 0.014 and 29.2% vs. 70.8%, *p* = 0.000).

### 3.2. The Societal Criticism for the Self-Testing and Its Parameters

About one third of the population-based sample characterized self-tests reliable and effective, and one quarter considered them as difficult and distressing, with no significant differences between genders. Two thirds reported self-tests to be expensive and three fifths considered them difficult to perform, again with no significant differences between genders. Vaccinated women were more likely to buy a self-test compared to men (63.6% vs. 54.9%, *p* = 0.029). Self-tests were considered more reliable and effective but distressing for the youth and more dangerous for the older generations. About half of the Baby Boomers disclosed that they would take drugs by themselves after a positive self-test. In addition, people who disclosed having a university education were more likely than the others declaring only basic schooling to estimate self-tests as reliable (34% vs. 25.8%, *p* = 0.032), effective (37.1% vs. 27.9%, *p* = 0.020) and less dangerous (50.2% vs. 40.3%, *p* = 0.016), and they reported they would follow further self-treatment at a lower percentage (31.7% vs. 41%, *p* = 0.019). The following Table 2 demonstrates descriptive statistics for the attitudes and societal criticism for self-testing’s parameters and issues.

Moreover, and concerning the environmental parameters, 74.8% throw the self-test waste to the rubbish bin, 9.4% to the recycle bin and 15.8% take them back to pharmacy, with no statistically significant differences among genders and education levels, but the last was mostly mentioned by Generation X and Baby Boomers. Furthermore, 23.6% wear gloves during autodiagnosis, mainly reported by older generations. In addition, 13.4% reported to have interacted with the liquid substances and buffers of the self-test, mostly the youth. One third of the population-based sample reported performing autodiagnosis in their dining room, the same percentage did so in the bathroom and 20.6% in the kitchen, and, finally, 81.3% seem to recognize the environmental burden of the waste of self-test cassettes, without any statistical significance between genders, generations and education levels.

### 3.3. The Societal Criticism for the Hospital Diagnosis and Its Issues

Table 3 shows descriptive statistics for the societal criticism about paralleled issues from autodiagnosis to hospital diagnosis.

About one half of the population-based sample considered EDs’ equipment and their staff as poor, their management and reliability rates were about the same and a fear of in-hospital SARS-CoV-2 infection was higher in women (82.9% vs. 74.3%, *p* = 0.009). Except management, all the other issues were statistically significant between education levels, and university education levels recorded the higher positive rates for EDs’ criticism. In addition, a positive reliability, effectiveness and a high danger’s rates were recorded, mainly from the youth.

### 3.4. Investigation of Potential Predictors for a Volition to Self-Testing and Emergency Department Visit

In total, 21.8% of the population-based sample declared that they would buy a self-test for autodiagnosis purposes if they expressed COVID-19 symptoms or if they had interacted with an identified case of COVID-19. A final logistic regression analysis, considering the volition to buy a self-test if needed as a dependent variable, found that reliability (OR 1.335; CI 0.060–0.300; *p* = 0.000) and danger (OR 5.068; CI 3139–8184; *p* = 0.000) had a significant impact on people’s consideration to buy a self-test (Table 4).

Furthermore, 30.1% of the population-based sample declared that they would go to a hospital rather than a private physician if they expressed COVID-19 symptoms or if they interacted with an identified case of COVID-19. A final logistic regression analysis, considering the volition to go to the hospital as a dependent variable, found that reliability (OR 3.207; CI 1987–5182; *p* = 0.000) and EDs’ effectiveness (OR 3.506; CI 2167–5670; *p* = 0.000) had a significant impact on people’s willingness to go to the ED instead of a private physician, if needed (Table 5).

## 4. Discussion

Society’s trajectory of futuristic technological has wielded a remarkable impact on scientific medical advancements over the last few years. Most medical decisions are nowadays driven by diagnostic tests. The latest diagnostical strategies, shifting towards modernization, embrace the rapid diagnostic tools as an avant means of futuristic medical diagnosis, availing various terrains, such as self-testing purposes. Except from critical cases requiring urgent diagnosis, autodiagnosis is the most important field, as an early detection of a medical condition provides better outcomes for the patient, the possible needed isolation or potential savings to the healthcare system. In such manner, autodiagnosis seems to be a demand, especially for epidemics or pandemic eras of infectious diseases, such as COVID-19.

Reliability seems to be a crucial issue for all people and in significant rates, except from the youth’s lower records, and was found to have a statistically significant impact on people’s volition to buy and perform a self-test. However, self-tests are rapid antigen tests that show a better diagnostic performance especially in cases with high viral loads [10,14,15]. As a result, falsely used rapid tests such as in autodiagnosis for non-exposed to SARS-CoV-2 individuals can lead to false results [10]. Thus, healthy people forced to perform a self-test but with a false positive result, may lose respect for the value of a future correctly utilized self-test. Retrospectively, people with symptomatic COVID-19 cases that were found negative in a self-test but tested positive in a further nucleic acid amplification test may generally consider rapid antigen tests as unreliable sources. In contrast to autodiagnosis, the reliability of ED diagnosis had a significant criticism that impacted people’s final willingness to visit a hospital ED if expressing COVID-19 symptoms. However, hospitals perform PCR assays in symptomatic individuals that are generally believed to be the gold standard, but false-negative test results, contrasted to false-positive ones, abound [15,16]. In addition, several other issues in imaging data or biochemical markers can affect the prompt diagnosis of COVID-19 in the ED [17]. In this way, ED diagnosis can be risky, and people who know falsely diagnosed individuals may disrespect the overall ED’s performance. In addition, alternative testing, such as anti-SARS-CoV-2 antibody tests, can lead to false-positive or false-negative test results for several reasons, which affects the final diagnosis [10]. Importantly, hospital reliability was highly estimated in the youth who may not have visited the ED several times as the other generations have. As a result, more accurate antigen, antibody or nucleic acid tests should be available for people and hospitals, for example, assays that distinguish several viruses’ genes in one performance, for a more reliable and accurate pathogen’s diagnosis.

Some people may need to buy and perform self-tests for several reasons, therefore considering them as expensive. Lessening the cost of rapid tests is a demand, not only for low-income countries but also for countries with financial crises, such as Greece, which has been devastated for a continuing decade by the financial crisis [18]. However, reducing the price of rapid tests may lessen their effectiveness or increase the rates of false tests. In addition, the difficulty of performing a rapid test may influence some people, especially kits demanding nasopharyngeal swabs; thus, easier tests can be produced, such as licking methods. Despite the fact that the danger of chemicals is minimized in the current devices and that poor devices have been recalled, about half of the participants considered them dangerous, and this issue played an important role in people’s volition to buy a self-test if needed. Misinformation for ethylene, as it is a carcinogen, may affect people’s consideration, but the sterilization process of medical devices is tightly controlled to ensure that any residue left over is negligible [19]. Moreover, public health misinformation about the correct use of self-tests can lead to a prolonged pandemic, since all COVID-19 symptomatic cases or those exposed to COVID-19 cases but not yet symptomatic should be tested, according to CDC recommendations [20]. Yet, the process of autodiagnosis may be stressful for some individuals, or it can lead to misinterpretation and confusion. In addition, self-treatment of self-recognized conditions with random or ineffective drugs can have a significant impact on individuals’ health [21]. Nevertheless, the level of education did not show any significance for autodiagnosis, as it did in criticism for EDs issues.

Another major issue concerning autodiagnosis via rapid tests is doubtlessly the environmental parameter. Plastic waste and toxic chemicals raise awareness for the environment during the COVID-19 pandemic, therefore, biodegradable biomedical manufacturing that are free of releasing toxic chemicals when they are incinerated must be considered [22]. Until then, it is a demand to inform the public to return self-test waste to the pharmacy, as well as wearing gloves during the process. In addition, people should be informed to perform autodiagnosis in safe places to avoid further indoor toxicity from plastics and buffers [23].

Hospitals are struggling with the COVID-19 pandemic, and especially the EDs face all the burden and the risk for the initial identification of COVID-19. Another study revealed the equipment limits, and especially the discrepancy rates for several medications in the ED, that were high even before the pandemic [24]. As a result, in several low-income countries or those facing a financial crisis, hospitals might not be well equipped for the COVID-19 pandemic. Furthermore, emergency physicians and other staff were facing the greatest risk of communicating with ill people or critical cases, and sad events such as violence and aggression in the ED can often be an overwhelming yet inevitable experience for staff, not to mention them during managing a pandemic [25]. However, more skilled physicians are required for front-line medical care, and more organized EDs and emergency clinics seem to be a demand for pandemic eras or infectious diseases, such as the university EDs, which are almost absent in Greece. In addition, ED staff are time-poor, and COVID-19 diagnosis requires urgent identification of the overall situation, from the medical history to the background of a case to biochemical markers or tests, for an accurate and effective diagnosis, especially for ambiguous yet critical cases [17,26]. A danger of catching SARS-CoV-2 in the ED was also revealed, especially from youth, and, broadly, the precise management in the ED recorded middle rates. Admittedly, the COVID-19 pandemic requires careful planning to facilitate the urgent restructuring of many aspects of an ED [27]. In times of increased patient loads during a pandemic wave, the need for strategies to manage patient flow in the ED has become increasingly important. ED senior management ought to plan, prepare, practice, review, analyze, assess and strategize for unexpected events, such as critical cases with false test results. Yet, undoubtedly, the quality of the leaders and managers of the ED regulate the environment where the ED staff can deliver the most effective, accurate and urgent medical care to their patients, and this is highly considered for people’s volition to visit a private doctor rather than an ED [28]. Finally, the emergency medicine field is doubtlessly evolving in our era, and thus accurate directions should be made in order to set prompt and precise ED facilities, physicians and patients’ management strategies.

## 5. Conclusions

The COVID-19 pandemic introduced massive rapid testing, especially for autodiagnosis purposes, in humanity. However, several issues regarding self-testing abound, and they make people reconsider buying a self-test, especially concerning the reliability or the potential danger—with environmental parameters and also the cost or the effectiveness being highly recorded. Hospital EDs faced the pandemic, and indubitably, they struggled while managing identified COVID-19 cases. Yet, the social questioning of the effectiveness and reliability of ED medical diagnosis was revealed from this study, and thus, more accurate and prompt strategies should be established from frontline physicians to manage diagnosis or false tests, since, in our decades, laboratory diagnosis is prevalent. It should not be forgotten that society recognizes and highlights the issues not only for autodiagnosis but for medical prognosis as well, and awareness for both diagnoses’ parameters should be raised for better and on-the-spot future diagnoses.

## Figures and Tables

**Table 1:** Genders and education level amongst generations, *n* = 614.

Generations	*n*	Genders		*p*-Value	Education Level		*p*-Value
		Male (% out of *n*)	Female (% out of *n*)		Basic School (% out of *n*)	BSc, MSc, PhD (% out of *n*)	
Generation Z	62	24 (38.7)	38 (61.3)	0.408	18 (29)	44 (71)	0.014
Millennials	216	107 (49.5)	107 (50.5)	0.200	63 (29.2)	153 (70.8)	0.000
Generation X	287	120 (41.8)	167 (58.2)	0.390	127 (44.3)	160 (55.7)	0.777
Baby Boomers	49	17 (34.7)	32 (65.3)	0.187	21 (42.9)	28 (57.1)	0.907

**Table 2:** Societal criticism for self-testing parameters and issues, *n* = 614.

Self-Test Criticism ^1^	Genders		*p*-Value	Generations				Education Level		*p*-Value
	Male (% within)	Female (% within)		Generation Z (% within)	Millennials (% within)	Generation X (% within)	Baby Boomers (% within)	Basic School (% within)	BSc, MSc, PhD (% within)	
Reliable	73 (27.2)	117 (33.8)	0.080	30 (48.4) ^a^	69 (31.9) ^a,b^	77 (26.8) ^b^	14 (28.6) ^a,b^	59 (25.8)	131 (34)	0.032
Expensive	183 (68.3)	244 (70.5)	0.550	40 (64.5) ^a^	150 (69.4) ^a^	201 (70) ^a^	36 (73.5) ^a^	166 (72.5)	261 (67.8)	0.221
Unmanageable	83 (24)	57 (21.3)	0.426	13 (21) ^a^	55 (25.5) ^a^	58 (20.2) ^a^	14 (28.6) ^a^	56 (75.5)	301 (78.2)	0.452
Dangerous	117 (43.7)	153 (44.2)	0.889	17 (27.4) ^a^	83 (38.4) ^a,b^	142 (49.5) ^b,c^	28 (57.1)	115 (50.2)	155 (40.3)	0.016
Distressing	66 (24.6)	104 (30.1)	0.136	32 (51.6) ^a^	57 (26.4) ^b^	69 (24) ^b^	12 (24.5) ^b^	58 (25.3)	112 (29.1)	0.314
Effective	81 (30.2)	126 (36.4)	0.121	31 (50) ^a^	76 (35.2) ^b^	85 (29.6) ^b^	15 (30.6) ^b^	64 (27.9)	143 (37.1)	0.020
Medication ^2^	97 (36.2)	119 (34.4)	0.670	15 (24.2) ^a^	60 (27.8) ^a^	118 (41.1) ^b^	23 (46.9) ^b^	94 (41)	122 (31.7)	0.019
Vaccination ^3^	147 (54.9)	220 (63.6)	0.029	44 (71) ^a^	134 (62) ^a^	162 (56.4) ^a^	27 (55.1) ^a^	136 (59.4)	231 (60)	0.881

^1^: Variables’ reported percentages refer to the sum of positive frequencies in 5-Likert type questions. ^2^: Medication issue refers to those who would risk for self-treatment after a positive self-test. ^3^: Vaccination issue refers to vaccinated who would buy a self-test after contacting a COVID-19 case. Each subscript letter a,b denotes a subset of age categories whose column proportions do not differ significantly from each other at the 0.05 level.

**Table 3:** Societal criticism for hospital EDs’ medical diagnosis parameters and issues, *n* = 614.

Emergency Departments’ Criticism ^1^	Genders		*p*-Value	Generations				Education Level		*p*-Value
	Male (% within)	Female (% within)		Generation Z (% within)	Millennials (% within)	Generation X (% within)	Baby Boomers (% within)	Basic School (% within)	BSc, MSc, PhD (% within)	
Equipment ^2^	114 (42.5)	158 (45.7)	0.439	22 (35.5) ^a^	93 (42.1) ^a^	135 (47) ^a^	22 (44.9) ^a^	89 (38.9)	183 (47.5)	0.037
Physicians ^2^	128 (47)	168 (48.8)	0.705	28 (45.2) ^a^	111 (51.4) ^a^	136 (47.4) ^a^	19 (38.8) ^a^	96 (58.1)	198 (51.4)	0.023
Reliability	130 (48.5)	171 (49.4)	0.822	37 (59.7) ^a,b^	121 (56) ^b^	121 (42.2) ^a^	22 (44.9) ^a,b^	95 (41.5)	206 (53.5)	0.004
Management	116 (43.3)	161 (46.5)	0.422	36 (58.1) ^a^	100 (46.3) ^a^	122 (42.5) ^a^	19 (38.8) ^a^	93 (40.6)	184 (47.8)	0.084
Effectiveness	128 (47.8)	173 (50)	0.582	40 (64.5) ^a^	106 (49.1) ^a,b^	132 (46) ^b^	23 (46.9) ^b^	92 (40.2)	209 (54.3)	0.001
Danger ^3^	199 (74.3)	287 (82.9)	0.009	56 (90.3) ^a^	178 (82.4) ^a,b^	213 (74.2) ^b^	39 (79.6) ^a,b^	165 (72.1)	321 (83.4)	0.001

^1^: Variables’ reported percentages refer to the sum of positive frequencies in 5-likert type questions. ^2^: Equipment and physicians’ issue refers to the adequate equipment and staff in EDs. ^3^: Danger issue refers to those who estimate EDs dangerous to catch a SARS-CoV-2 infection. Each subscript letter a,b denotes a subset of age categories whose column proportions do not differ significantly from each other at the 0.05 level.

**Table 4:** Logistic regression with a volition to buy a self-test if needed as a dependent variable.

Variables	B	S.E.	Wald	df	Sig.	Exp(B)	95% C.I. for EXP (B)	
							Lower	Upper
Basic school	−0.169	0.225	0.561	1	0.454	1.184	0.761	1.841
Reliability	2.004	0.409	24.011	1	0.000	1.335	0.060	0.300
Danger	−1.623	0.244	44.080	1	0.000	5.068	3.139	8.184
Cost	−0.284	0.266	1.134	1	0.287	1.328	0.788	2.239

**Table 5:** Logistic regression with a volition to go to the hospital ED if needed as a dependent variable.

Variables	B	S.E.	Wald	df	Sig.	Exp(B)	95% C.I. for EXP (B)	
							Lower	Upper
University education	0.267	0.212	1.584	1	0.208	1.306	0.862	1.978
Reliability	1.165	0.245	22.653	1	0.000	3.207	1.987	5.182
Effectiveness	1.254	0.245	26.144	1	0.000	3.506	2.167	5.670
Danger	0.111	0.258	0.185	1	0.667	1.118	0.674	1.854

## Data Availability

Data sharing is not applicable to this article. The data are not publicly available due to restrictions. They contain information that could compromise the privacy of the participants.
